# Spatial Analysis on Supply and Demand of Adult Surgical Masks in Taipei Metropolitan Areas in the Early Phase of the COVID-19 Pandemic

**DOI:** 10.3390/ijerph19116704

**Published:** 2022-05-31

**Authors:** Chien-Chou Chen, Guo-Jun Lo, Ta-Chien Chan

**Affiliations:** 1 Department of Statistics and Information Science, Fu Jen Catholic University, New Taipei City 242, Taiwan; tojoechen@gmail.com (C.-C.C.); e280331@gmail.com (G.-J.L.); 2 Research Center for Humanities and Social Sciences, Academia Sinica, Taipei 115, Taiwan; 3 Institute of Public Health, School of Medicine, National Yang Ming Chiao Tung University, Taipei 112, Taiwan

**Keywords:** Bayesian hierarchical modeling, Voronoi diagram, surgical mask, small area estimation, supply and demand

## Abstract

This study aimed to assess the gap between the supply and demand of adult surgical masks under limited resources. Owing to the implementation of the real-name mask rationing system, the historical inventory data of aggregated mask consumption in a pharmacy during the early period of the COVID-19 outbreak (April and May 2020) in Taiwan were analyzed for supply-side analysis. We applied the Voronoi diagram and areal interpolation methods to delineate the average supply of customer counts from a pharmacy to a village (administrative level). On the other hand, the expected number of demand counts was estimated from the population data. The relative risk (RR) of supply, which is the average number of adults served per day divided by the expected number in a village, was modeled under a Bayesian hierarchical framework, including Poisson, negative binomial, Poisson spatial, and negative binomial spatial models. We observed that the number of pharmacies in a village is associated with an increasing supply, whereas the median annual per capita income of the village has an inverse relationship. Regarding land use percentages, percentages of the residential and the mixed areas in a village are negatively associated, while the school area percentage is positively associated with the supply in the Poisson spatial model. The corresponding uncertainty measurement: villages where the probability exceeds the risk of undersupply, that is, Pr (RR < 1), were also identified. The findings of the study may help health authorities to evaluate the spatial allocation of anti-epidemic resources, such as masks and rapid test kits, in small areas while identifying priority areas with the suspicion of undersupply in the beginning stages of outbreaks.

## 1. Introduction

A scientific review of the literature offers evidence in support of masks used as a source to reduce the transmission of infectious diseases, such as coronavirus disease (COVID-19), caused by the severe acute respiratory syndrome coronavirus-2 [1]. In response to the COVID-19 pandemic, the Taiwanese government launched a nationwide real-name mask rationing system to distribute adult and child surgical masks to citizens in February 2020 [2], followed by the mandated wearing of masks when accessing public transportation in April 2020. Under the epidemic control policy, access to surgical masks was limited to community pharmacies and district public health centers across the country. Customers freely purchased masks at the above-mentioned vendors but were verified via their National Health Insurance cards [3]. Based on the weekly rationing number of masks, the quota of masks that each person could buy was predetermined from February to May until the Ministry of Health and Welfare (https://www.mohw.gov.tw/, accessed on 1 May 2022) announced that surgical masks could be freely sold in retail channels, such as convenience stores, on 1 June 2020 [4]. However, owing to its close geographic and economic ties with China, Taiwan was expected to have the second-highest number of imported cases in the early phase of the COVID-19 pandemic [5].

Small area estimation (SAE) leverages statistical techniques to estimate parameters of a particular demographic in a set of small geographical areas [6], which provides insights for reorganizing local productive processes [7], understanding the extent of household poverty in districts [8], and portraying the progress of the HIV prevalence rate in districts [9]. A model-based SAE incorporates random effect terms in a mixed model to borrow information from neighboring areas when estimating spatially correlated random effects [10,11]. For non-normal autocorrelated responses, such as auto-Poisson counts, in SAE, there are no closed-form solutions for normalization factors [12] and methods such as Markov chain Monte Carlo procedures [13] are required, which are applied in Bayesian inference. Bayesian hierarchical modeling, in which parameter values can arise from distributions, is used to borrow strength across areas using both spatially structured and unstructured random effects to estimate cross-stratified counts of interest [14]. Furthermore, the use of Bayesian methodology has seen great advances because of the introduction of software and statistical packages such as WinBUGS [15] and the integrated nested Laplace approximation (INLA) package in R [16].

Serving as the fundamental administrative unit in Taiwan, a village area comprises rich aggerated demographic and economic information from a regular nationwide survey and is also the administrative unit for disease surveillance [17]. However, to our knowledge, few studies have revealed the supply demand mismatch of medical resources, incorporating SAE and small-area demographic and economic information [18,19]. We propose a generic method to estimate the supply and demand of customers residing in a small area (village), using an adult surgical mask as an example. The findings of the study may help health authorities evaluate the allocation of anti-epidemic resources at the village level and identify villages with suspected undersupply in the early phase of a pandemic.

## 2. Materials and Methods

This study aimed to model the supply (in terms of customer counts) of adult surgical masks served in a village in the Taipei metropolitan areas. In the following subsections, we highlight the following: (1) preprocessing of inventory datasets of adult surgical masks, (2) delineation and areal interpolation of the customer count of adult surgical masks using a Voronoi diagram from a pharmacy (source zone) to a village (target zone), and (3) specification of a Bayesian spatial modeling framework on the relative risk (RR) of supply.

### 2.1. Data and Preprocess of Data

**Figure 1:**
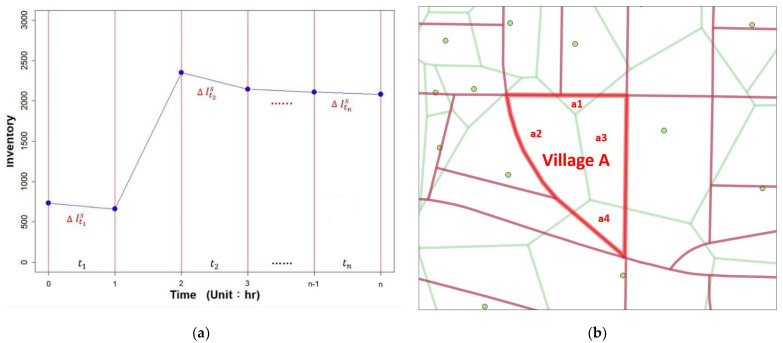
(**a**) Preprocessing the inventory datasets of adult surgical masks in a pharmacy; (**b**) areal interpolation of the supply from Voronoi diagrams (source zone, colored in green) to villages (target zone, colored in red). (For example, village A = a1 + a2 + a3 + a4).

Inventory datasets for surgical masks were obtained from the data market platform of the National Center for High-Performance Computing (https://scidm.nchc.org.tw/, accessed on 1 May 2022). The historical inventory number of aggregated adult and children surgical masks per 10 min between 9 April 2020, and 27 May 2020, for pharmacies located in Taipei City and New Taipei City was included (https://scidm.nchc.org.tw/dataset/nhi-maskdata-archive, accessed on 1 May 2022) [20]. To obtain the daily average customer number served by each pharmacy, we (1) examined the inventory number of adult surgical masks to identify the negative slope of inventory intervals (∆$I_{t1}^{s}$…, i.e., the change in stock level) every hour, (2) summed up the net inventory values of *n* negative slope intervals (∆$I_{t1}^{s}$ + ∆$I_{t2}^{s}$ + … + ∆$I_{tn}^{s}$) (Figure 1a) to obtain the total number of adult surgical mask in date *t* ($=I_{t}^{s}$) for store *s*, (3) calculated the average sales number per day (𝐼^𝑠^ = $\frac{\sum^{\;}I_{t}^{s}}{T}$) for store *s* in a total of *T* days, and (4) estimated the average customer served per day (*=*$\frac{I^{s}}{9}$) for store *s* since the predetermined quotas of adult surgical masks per person per 2-weeks were nine masks [21] at that period under the real-name mask rationing system in Taiwan [4].

### 2.2. Voronoi Diagram and Areal Interpolation

This study aimed to estimate the daily average number of service counts at the village level. In Section 2.1, we demonstrated the estimation of the average number of service counts per day for each pharmacy. The original datasets of supply counts were at the point level (pharmacy), whereas demand counts were extracted from the areal level (village). Therefore, methods such as the Voronoi diagram and areal interpolation that deal with the change-of-support problem [22], that is, spatial misalignment between supply and demand, are required. We constructed the Voronoi diagram [23], which partitions a set of points into areas for each pharmacy to generate a mutually exclusive surface of service counts. Next, we applied areal interpolation [24] methods to redistribute the service counts from the Voronoi diagram (source zone colored in green, and the centroid is the location of the pharmacy) to the village (target zone colored in red) under the assumption of homogeneous population density [25] (Figure 1b). For example, the total customer counts of village A (thick red lines) are summed from four subsets (intersects): a1, a2, a3, and a4.

### 2.3. Bayesian Spatial Modeling on the Supply of Adult Surgical Mask

We propose four Bayesian hierarchical models, namely Poisson, negative binomial, Poisson spatial, and negative binomial spatial models, to investigate the RR of supply. For the *i*th village, the number of served customer count 𝑌_𝑖_ (Formula (1)) is modeled as supply following a Poisson distribution with mean 𝜆_𝑖_ [26]:

𝑌_𝑖_ ~ 𝑃𝑜𝑖𝑠𝑠𝑜𝑛(𝜆_𝑖_ = 𝐸_𝑖_𝜌_𝑖_) (1)

log(𝜌_𝑖_ = 𝜆_𝑖_/𝐸_𝑖_) = 𝑏_0_ + 𝑏_1_𝑥_1_ + ⋯ + 𝑏_6_𝑥_6_ + 𝑢_𝑖_ + 𝜈_𝑖_ (2)

𝜆_𝑖_ = 𝐸_𝑖_𝜌_𝑖_ where 𝐸_𝑖,_ treated as an offset (= 𝑟 × 𝑃𝑜𝑝_𝑖_), is the expected number (demand) of served adults for each village *i*. 𝑟 (= ∑𝑌_𝑖_/∑𝑃𝑜𝑝_𝑖_) is the observed (overall) service rate for the total *n* villages [27] and 𝑃𝑜𝑝_𝑖_ is the adult number (aged ≥ 15 years) in village *i*. 𝜌_𝑖_ is the village-specific RR that quantifies whether the village *i* has an over- (𝜌_𝑖_ = 𝜆_𝑖_/𝐸_𝑖_ > 1) or under- (𝜌_𝑖_ = 𝜆_𝑖_/𝐸_𝑖_ < 1) supply than that expected. 𝑏_0_ is the intercept, representing the average RR in the study area. 𝑏_1_,…, 𝑏_6_ are the estimated parameters for independent variable 𝑥_1_,…, 𝑥_6_ respectively, including: (1) the total number of pharmacy in a village, (2) the median annual per capita income in 2019 of the village, the percentages of (3) business area, (4) residential area, (5) mixed area, and (6) school area in a village.

To borrow strength from surrounding villages, random effect terms: 𝑢_𝑖_ + 𝜈_𝑖_ were added after the fix effect terms (𝑏_0_ + 𝑏_1_𝑥_1_ + ⋯ + 𝑏_6_𝑥_6_) and defined as the Poisson spatial model (Formula (2)). 𝑢_𝑖_ is the spatially structured residual using intrinsic conditional autoregressive (ICAR) specification, that is, the Besag-York-Mollie model [28] such that:

(3) $${𝑢}_{𝑖}({𝑢}_{-𝑖}\sim \;𝑁𝑜𝑟𝑚𝑎𝑙\;({𝜇}_{𝑖}+\frac{1}{N_{i}}{\sum^{\;}}_{j=1}^{n}𝑎{𝑖}_{𝑗}\;({𝑢}_{𝑗}-{𝜇}_{𝑗}),\;{{𝑠}_{𝑖}}^{2})$$

where 𝜇_𝑖_ is the mean for village *i* and 𝑠_𝑖_^2^ = 𝜎*_u_*^2^ / 𝑁_𝑖_ is the variance for village *i,* which depends on the number of neighbors 𝑁_𝑖_. 𝑎_𝑖𝑗_ is the weighting matrix defining the contiguity of villages (Formula (3)). 𝜈_𝑖_ is the unstructured residual such that (Formula (4)):

𝜈_𝑖_ ~ 𝑁𝑜𝑟𝑚𝑎𝑙 (0, 𝜎_𝜈_^2^) (4)

For a Poisson distribution, we assume V(𝜆_𝑖_) = E(𝜆_𝑖_). Considering overdispersion data: V(𝜆_𝑖_) > E(𝜆_𝑖_), a Poisson distribution with a Gamma prior: Gamma (𝜗, 𝜗) on 𝜆_𝑖_, that is, a negative binomial distribution (Formula (5)), might be suggested [29,30]:

(5) $$𝑌{𝑖}_{\;}\sim \;𝑁𝑒𝑔𝑎𝑡𝑖𝑣𝑒𝐵𝑖𝑛𝑜𝑚𝑖𝑎𝑙\;({𝜆}_{𝑖},\;{𝜆}_{𝑖}+\frac{\lambda_{i}^{2}}{\vartheta })$$

Additionally, identical random effect terms from Formula (2) were specified for the negative binomial spatial model. In the absence of information, default vague priors are applied to the fixed effect. Regarding the prior distributions of random effects, a gamma (0.0001, 0.0001) prior with small values of precision parameters was chosen for both structural and unistructural random effects [31,32]. In addition to estimate the 𝜌_𝑖_, we further calculated the exceedance probabilities of RR of supply being less than 1, that is, Pr (RR < 1) [33] to identity the village with suspicion of undersupply.

The Poisson and negative binormal models serves as the benchmark for their corresponding spatial counterparts, and to investigate the performance of these models, the deviance information criterion (DIC) [34,35] and Watanabe-Akaike information criterion (WAIC) [34,36] were evaluated. For predictive accuracy assessment, the mean absolute percentage error (MAPE) and mean squared error (MSE) were also applied [37]. Bayesian hierarchical modeling was implemented using the INLA approach [38], which is more reliable than the harmonic mean method [39], in the R-INLA R package [40,41].

## 3. Results

We modelled the supply and demand of adult surgical masks served at the village level in Taipei metropolitan areas using inventory datasets during the early phase of the COVID-19 pandemic. The RR of supply, which is the average number of adults served per day divided by the expected number in a village, was modelled using a Bayesian hierarchical framework. The corresponding uncertainty measurement, villages where the probability exceeds the risk of undersupply, that is, Pr (RR < 1), was also presented.

**Figure 2:**
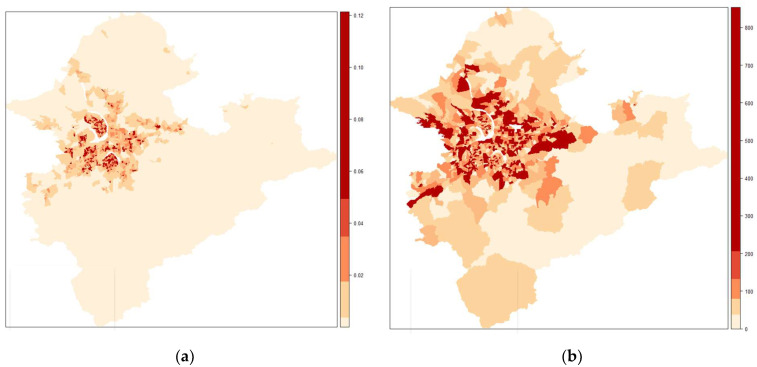
(**a**) Population density (*#people/meter*^2^) of study area at the village level (*n* = 1488); (**b**) average daily supply (customer counts) of adult surgical masks at the village level in Taipei metropolitan areas.

As the largest metropolis in Taiwan, the Taipei metropolitan areas encompass a population of 6.59 million and a mean population density of 0.002 (*#pop/meter*^2^) (Figure 1a) in 2020. There are a total of 1488 villages and 1774 pharmacies in the study area. The median population of the villages is approximately 4268 people. The median supply of adult surgical masks, i.e., the average daily customer count in a village, is 105 adults (Figure 2b; Table 1), whereas the total supply in the study area is 193,052 customers, indicating an overall service rate of *r* = 0.033.

Figure 2a shows the 2020 population density of Taipei metropolitan areas, in which high population density values are concentrated in central business district areas (colored in red). Ranging from 0 to 854 (Table 1), the distributions of the average daily supply counts of adult surgical masks in a village were (presented in Figure 2b) were similar to the pattern of population density (Figure 2a).

The median values for the independent variables include (1) the total number of pharmacies in a village, (2) the median annual per capita income of the village, the percentages of (3) business, (4) residential, (5) mixed, and (6) school areas in a village are 1, 439,000 (New Taiwan Dollars), 0.019, 0.165, 0.127, and 0.001, respectively. The raw RR, which ranged from 0 to 18.896, had a median value of 0.850. (Table 1). To rule out multicollinearity among the independent variables, the values of the variance inflation factors (VIF) for the six variables were also reported (Table 1). The VIF values were all less than 10 [42], indicating that multicollinearity was not observed. The size parameter 𝜗 for the negative binomial model is 1.600 (Table 2), implying overdispersion in the data.

For the Poisson spatial model (Table 2), we observed that the posterior mean of the independent variable: store number is $\hat{\;\beta }$_𝑠𝑡𝑜𝑟𝑒𝑁𝑢𝑚𝑏𝑒𝑟_ = 0.124 with a 95% credible interval equals to (0.095, 0.153) (Table 3), indicating the store number increases the supply. On the contrary, $\hat{\;\beta }$_log(__𝑀𝑒𝑑𝑖𝑎𝑛𝐼𝑛𝑐𝑜𝑚𝑒)_ = −0.565(−0.853, −0.277), $\hat{\;\beta }$_𝑟𝑒𝑠𝑖𝑑𝑒𝑛𝑡𝑖𝑎𝑙𝐴𝑟𝑒𝑎%_ = −2.821(−3.224, −2.418) and $\hat{\;\beta }$_𝑚𝑖𝑥𝐴𝑟𝑒𝑎%_ = −0.990(−1.491, −0.489) have an inverse relationship on the supply. Conversely, $\hat{\;\beta }$_𝑠𝑐ℎ__𝑜𝑜𝑙𝐴𝑟𝑒𝑎%_ = 0.467(0.012, 0.923) is positively associated with the supply. Regarding the random effect, the corresponding posterior means (standard deviations) of spatial (1/𝜎*_u_*^2^) and nonspatial (1/𝜎_𝜈_^2^) parameters for the Poisson spatial model are 0.497 (0.048) and 6.380 (1.279), respectively (Table 2). For the negative binomial spatial model, since the size parameter 𝜗 is large (posterior mean = 24.957), the values of parameter are similar to those of the Poisson spatial model since the variance of the negative binomial spatial model is approaching to its mean.

**Figure 3:**
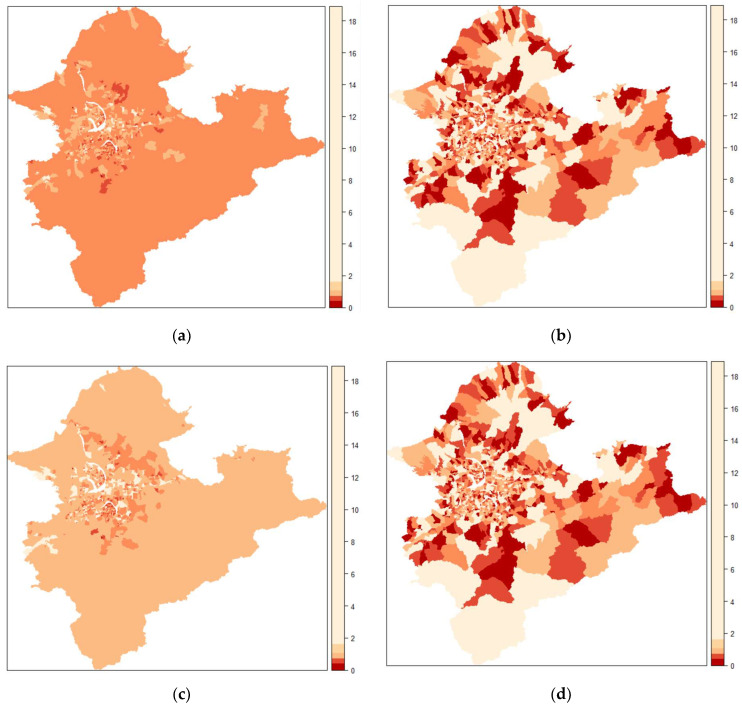

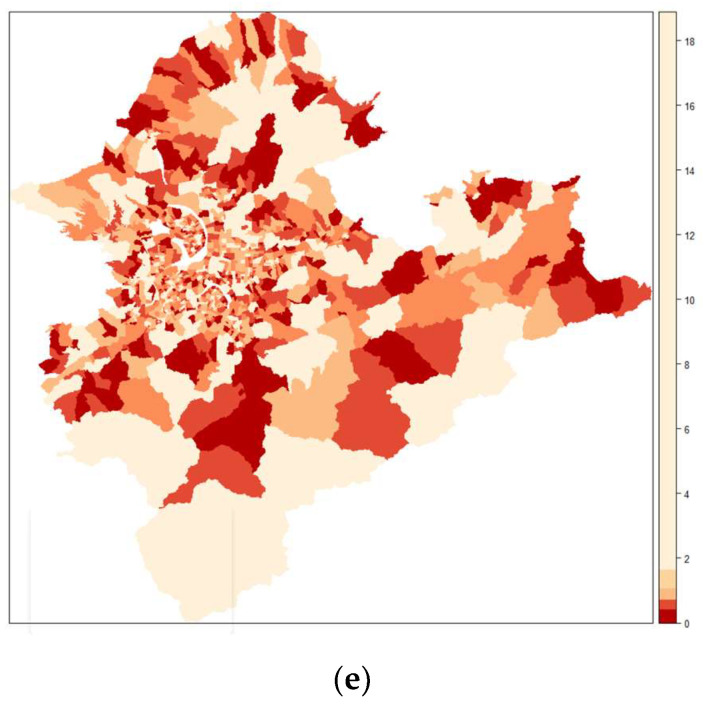
Comparison of four different Bayesian hierarchical models: (**a**) Poisson, (**b**) Poisson spatial, (**c**) negative binomial, and (**d**) negative binomial spatial on the relative risks, the observed number of adults served divided by the expected number of adults served, of the supply counts of adult surgical masks against the raw relative risks (**e**) by village in Taipei metropolitan areas.

**Figure 4:**
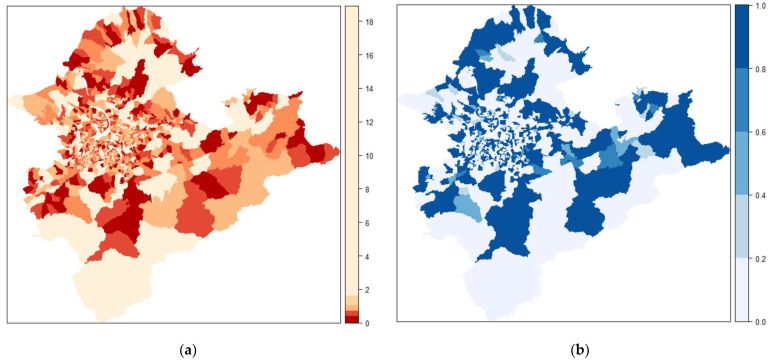
(**a**) Relative risks (RRs) of the Poisson spatial model on the supply counts of adult surgical masks; (**b**) the corresponding exceedance probability of undersupply: Pr (RR < 1).

We suggest a Poisson spatial model based on DIC, WAIC, MAPE, and MSE (Table 2), with corresponding values of 12,240, 11,876, 0.035, and 0.002, respectively, with the best goodness of fit. Figure 3a–d show village-specific maps of the estimated RRs of supply counts based on four Bayesian hierarchical models (Poisson, Poisson spatial, negative binomial, and negative binomial spatial models, respectively) against the raw RRs (Figure 3e). Overall, we observed that aspatial models (Figure 3a,c) underestimate the RRs, and the patterns agreed with the results of the goodness of fit tests in Table 2. Finally, we calculated the exceedance probabilities of the RRs of supply being less than 1, that is, Pr (RR < 1) (Figure 4b) for the Poisson spatial model (Figure 4a), which is useful for identifying villages with an undersupply.

## 4. Discussion

This study proposed a generic method for estimating the supply and demand of adult surgical mask customers served in a small area. Demand is characterized by the expected number of customers served, whereas the supply side estimate comes from the observed number of customers served in a village, delineated from the neighboring pharmacies. The under- or over-supply of surgical mask in a village of Taipei metropolitan areas is evaluated by 𝜌_𝑖_ < 1 or 𝜌_𝑖_ > 1, which is defined as the RR in the literature of Disease Mapping and Spatial Epidemiology [43,44].

Taking a surgical mask as an example, we identified villages with a suspected undersupply of adult surgical masks (Figure 4b). We also evaluated the factors associated with the supply of customers in the ecological regression (Table 3). The findings of this study may be helpful for health authorities to evaluate the spatial allocation of anti-epidemic products, such as vaccines, in small areas while identifying priority communities with an undersupply [45,46].

We observed that the number of pharmacies in a village is associated with increasing supply, while the median annual per capita income has an inverse relationship in the Poisson spatial model. Regarding land use percentages, the percentages of residential and mixed areas in a village were negatively associated, while the percentage of school area was positively associated with supply. In a spatial dependency model analyzing the sales of medical products [47], a significant negative sign on the median household income (block group level) was identified in Houston, Texas. Meanwhile, land use mix, defined as the entropy index of residential, commercial, business, public, cultural, and other land use areas against the total floor area, is positively associated with retail sales in Seoul, South Korea [48].

Similar to the study assessing offenders’ counts across the enumeration districts of Sheffield, England in 1995, we found that compared to the negative binomial counterpart, the Poisson spatial model provides better model fitting, indicating that all the variance excess is successfully modeled by the random effect term [49].

The timely distribution of anti-epidemic materials is crucial in the early stages of an outbreak, such as COVID-19. Owing to the implementation of the real-name mask rationing system, historical inventory data of aggregated mask consumption during the early period of the COVID-19 outbreak in Taiwan was recorded for supply side analysis. We applied the Voronoi diagram and areal interpolation methods to delineate the average supply count from a store to a village. Since the variance of the average sales count for each store was also estimated, further evaluation of the uncertainty of the average sales count for each village can be performed by Monte Carlo simulation [50]. For the demand side estimation, we implemented the overall service rate 𝑟 to each village. Indirect standardization accounting for population size, age, and gender could be applied for the estimation of cross-stratified 𝑟^𝑎𝑔𝑒^^×^^𝑔𝑒𝑛𝑑𝑒𝑟^ to obtain 𝐸_𝑖_ = ∑ *r*^𝑎𝑔𝑒×^^𝑔𝑒𝑛𝑑𝑒𝑟^ × 𝑝𝑜𝑝_𝑖_^𝑎𝑔𝑒^^×^^𝑔𝑒𝑛𝑑𝑒𝑟^ [51], if cross-stratified counts (age by gender strata) of inventory data are available from the Ministry of Health and Welfare in the future.

The limitations of this study include the following: (1) we studied pharmacies located in Taipei metropolitan areas, and our findings should be generalized to other locations with caution; (2) due to the unavailability of commuting flow datasets at the village level, the effect of in-/out- flows on the consumption of surgical masks was ignored; (3) we assumed a homogeneous population density when conducting areal interpolation of supply counts, which could be released by methods such as dasymetric mapping [52] when additional ancillary information is available; (4) individual differences in consumption (such as knowledge of hygiene, socioeconomic status, and purchasing power) and reuse of surgical masks [53,54] were ignored in this ecological study; (5) we did not consider the irregular change in stock level resulting from the logistics system, and such information can be extracted and included in the model for adjustment.

## 5. Conclusions

Under the Bayesian hierarchical framework, we proposed a generic method to estimate the supply and demand of customer counts at the village level in Taipei metropolitan areas during the initial stages of a pandemic, using adult surgical masks as an example. The findings of this study may be helpful for health authorities to evaluate the spatial allocation of anti-epidemic products, such as masks and rapid test kits, in small areas, while identifying priority areas with suspected undersupply.

## Figures and Tables

**Table 1:** Descriptive statistics of the independent/dependent variables (supply count) and raw relative risks 𝜌_𝑖_ (*n* = 1488).

Variable	Minimum	1st Quantile	Median	3rd Quantile	Maximum	VIF
store number	0	0	1	2	9	1.136
median income (1000 NTD)	222	378	439	528	1031	1.076
business area %	0	0.006	0.019	0.052	0.457	1.118
residential area %	0	0.062	0.165	0.287	0.783	1.197
mixed area %	0	0.024	0.127	0.233	0.735	1.260
school area %	0	0	0.001	0.031	0.632	1.040
supply count	0	47	105	186	854	−
𝜌_𝑖_	0	0.491	0.850	1.446	18.896	−

VIF: variance inflation factor.

**Table 2:** Posterior means and standard deviations (sd) for four Bayesian hierarchical models.

Variable	Poisson: Mean(sd)	Negative Binomial: Mean(sd)	Poisson Spatial: Mean(sd)	Negative Binomial Spatial: Mean(sd)
intercept	1.099(0.062)	2.190(0.564)	3.702(0.900)	3.718(0.906)
store number	0.119(0.001)	0.137(0.016)	0.124(0.015)	0.124(0.015)
log(median income)	−0.214(0.010	−0.341(0.094)	−0.565(0.147)	−0.565(0.147)
business area %	1.632(0.039)	1.833(0.410)	0.559(0.411)	0.559(0.413)
residential area %	−0.976(0.021)	−1.607(0.167)	−2.821(0.205)	−2.822(0.207)
mixed area %	0.426(0.021)	0.131(0.214)	−0.990(0.255)	−0.990(0.258)
school area %	1.219(0.025)	0.939(0.232)	0.467(0.232)	0.467(0.234)
spatial component (1/𝜎*_u_*^2^)			0.497(0.048)	0.508(0.042)
iid component (1/𝜎_𝜈_^2^)			6.380(1.279)	7.689(1.715)
size parameter (𝜗)		1.600(0.055)		24.957(9.606)
DIC *	98,613	17,015	12,240	13,616
WAIC *	97,560	17,019	11,876	13,891
MAPE *	0.694	0.600	0.035	0.066
MSE *	1.738	1.671	0.002	0.039

* DIC, deviance information criterion; WAIC, Watanabe-Akaike information criterion; MAPE, mean absolute percentage error; MSE, mean squared error.

**Table 3:** The 95% credible intervals of the posterior mean of the independent variables by four Bayesian hierarchical models.

Title 2	Variable	0.025 Quantile	0.500 Quantile	0.975 Quantile
Poisson	store number	0.116	0.119	0.121
	log(median income)	−0.234	−0.214	−0.194
	business area %	1.555	1.632	1.708
	residential area %	−0.016	−0.976	−0.936
	mixed area %	0.385	0.426	0.467
	school area %	1.170	1.219	1.268
Negative binomial	store Number	0.106	0.136	0.168
	log(median income)	−0.523	−0.340	−0.155
	business area %	1.041	1.828	2.649
	residential area %	−1.932	−1.607	−1.279
	mixed area %	−0.286	0.130	0.552
	school area %	0.492	0.936	1.401
Poisson spatial	store number	0.095	0.124	0.153
	log(median income)	−0.853	−0.565	−0.277
	business area %	−0.248	0.559	1.366
	residential area %	−3.224	−2.821	−2.418
	mixed area %	−1.491	−0.990	−0.489
	school area %	0.012	0.467	0.923
Negative binomial spatial	store number	0.095	0.124	0.153
	log(median income)	−0.853	−0.565	−0.275
	business area %	−0.251	0.559	1.372
	residential area %	−3.228	−2.823	−2.416
	mixed area %	−1.495	−0.990	−0.482
	school area %	0.009	0.466	0.926

## Data Availability

Raw data of this study are openly available from the data market platform of the National Center for High-performance Computing, Taiwan (https://scidm.nchc.org.tw/, accessed on 1 May 2022).
